# 

**Published:** 1817-01

**Authors:** 


### THE LONDON MEDICAL AND PHYSICAL JOURNAL;

CONTAINING

ORIGINAL CORRESPONDENCE of EMINENT PRACTITIONERS,

AND THE

EARLIEST INFORMATION ON SUBJECTS CONNECTED WITH

MEDICINE, SURGERY, CHEMISTRY, PHARMACY, BOTANY, AND NATURAL HISTORY.

*Successively conducted by*

T. BRADLEY, M.D.

A. F. M. WILLICH, M.D.
R. BATTY, M.D.
A. A. NOEHDEN, M.D.
J. ARNEMAN, M.D.
J. P. PFAFF, M.D.
J. ADAMS, M.D.
W. SHEARMAN, M.D.
W. ROYSTON, Esq.

AND

SAMUEL FOTHERGILL, M.D.

Assisted by eminent Gentlemen, in the various Branches of the Profession.

VOL. XXXVII. 5143

FROM JANUARY TO JUNE, 1817.

Et quoniam variant morbi, variabimus artes;

Mille mali species, mille salutis erunt.

*LONDON:*

PRINTED FOR THE PROPRIETORS, BY J. ADLARD, 23, BARTHOLOMEW-CLOSE, AND 39, DUKE-STREET, SMITHFIELD.

PUBLISHED BY J. SOUTER, NO. 1, PATERNOSTER-ROW; AND MAY BE HAD OF ALL BOOKSELLERS.

W1
LO 31

### BOOKS PREPARING FOR PUBLICATION.

Dr. Burrows, of Gower-street, is preparing for the press a volume of "Commentaries on Mental Derangement."

In a few days will be published, a second edition of the Institutions of Physiology, by Professor Blumenbach; with extensive Notes, by John Elliotson, M.D.

Shortly will be published, a new edition of the late Mr. Saunders's work on the Anatomy and Diseases of the Ear. This edition will be of the same size as the Treatise on the Eye.

In the press, a miniature edition of the late Dr. Denman's Aphorisms on the use of the Forceps and Vectis; with a correct portrait of the author.

Speedily will be published, An Inquiry into the Effects of Spirituous Liquors on the Physical and Moral Faculties of Man, and on the Happiness of Society.

Mr. Samuel Young, in the course of the spring, will publish a further Report of Cancerous Cases successfully treated by the means of Mechanical Pressure.

---

### CATALOGUE OF MEDICAL BOOKS.

A Practical Synopsis of Cutaneous Diseases, according to the Arrangement of Dr. Willan; exhibiting a concise View of the Diagnostic Symptoms, and the Method of Treatment. By Thomas Bateman, M.D. F.R.S. Physician to the Public Dispensatory, and to the Fever Institution. Fourth Edition. 8vo. Plates.

Observations on the Structure of the Brain; comprising an Estimate of the Claims of Drs. Gall and Spurzheim to discovery in the Anatomy of that Organ. By John Gordon, M.D. F.R.S. in Edinburgh, &c. &c.

---

### *NOTICES TO CORRESPONDENTS.*

*We do not recollect to have seen the Publication mentioned by* L. G.—Mr. Yeatman's *paper shall be inserted as soon as his drawing is engraved.*—*Both* Mr. Kerr's *Letters are received, and his instructions attended to.*—Sceptic's *Letter would not suit the generality of our readers; we can, however, inform him, that soft soap only differs from hard in the first being made of vegetable, the latter of fossil alkali; or, to use a more common language, soft soap is made of potash, hard of soda. The soft may be the best for washing off infectious matter; but neither of them can be depended on.*

*We have received, and shall insert in our next Number, a most interesting account of the last illness, and appearances after death, of our esteemed quandam fellow-labourer,* Mr. Royston.

*Communications are received from* Drs. Sutton *and* Yeats; Messrs. Wm. Hey, Sandford, Smerdon, R. N. Starr, Ward, F. H., *and from a* Young but Constant Reader, *&c.; all of which shall appear in their order. We have also received a great number of foreign communications, some of no inconsiderable importance.*

